# Bioassay-Guided Evolution of Glycosylated Macrolide Antibiotics in Escherichia coli

**DOI:** 10.1371/journal.pbio.0050045

**Published:** 2007-02-06

**Authors:** Ho Young Lee, Chaitan Khosla

**Affiliations:** 1 Department of Chemistry, Stanford University, Stanford, California, United States of America; 2 Department of Chemical Engineering, Stanford University, Stanford, California, United States of America; Lawrence Berkeley National Laboratory, United States of America

## Abstract

Macrolide antibiotics such as erythromycin are clinically important polyketide natural products. We have engineered a recombinant strain of Escherichia coli that produces small but measurable quantities of the bioactive macrolide 6-deoxyerythromycin D. Bioassay-guided evolution of this strain led to the identification of an antibiotic-overproducing mutation in the mycarose biosynthesis and transfer pathway that was detectable via a colony-based screening assay. This high-throughput assay was then used to evolve second-generation mutants capable of enhanced precursor-directed biosynthesis of macrolide antibiotics. The availability of a screen for macrolide biosynthesis in E. coli offers a fundamentally new approach in dissecting modular megasynthase mechanisms as well as engineering antibiotics with novel pharmacological properties.

## Abstract 2

An erythromycin-producing*E. coli* recombinant strain with an antibiotic-overproducing mutation in the mycarose biosynthesis pathway produced second-generation mutants capable of directed biosynthesis of enhanced precursor macrolide antibiotics.

## Author Summary

The antibacterial activity of erythromycin, an important polyketide antibiotic precursor, requires the transfer of two unusual sugars called mycarose and desosamine (both glycosyl groups), onto the nonsugar part of the glycoside molecule (macrocyclic aglycone). We reconstituted the biosynthetic pathways of both sugars in Escherichia coli to yield the 6-deoxyerythromycin D antibiotic. By engineering a recombinant strain of E. coli that produces the bioactive macrolide 6-deoxyerythromycin D from propionate, we have developed a fundamentally new tool for enhancing the efficiency of biosynthetic engineering of this class of antibiotics. Initially, this recombinant strain produced barely enough antibiotic activity to establish an activity-based screening assay. We therefore used the assay to screen for antibiotic overproducers. After three rounds of screening, we identified E. coli cells that overproduced the 6-deoxyerythromycin D antibiotic with significant modifications in the mycarose biosynthetic pathway. We used the same activity-based screening system to evolve E. coli mutants capable of more efficient precursor-directed biosynthesis. As the first example of bioassay-guided evolution of an antibiotic pathway in E. coli, these results open the door for harnessing the power of genetics for mechanistic investigations into polyketide synthases and also for biosynthetic engineering.

## Introduction

Polyketides are a diverse and clinically important class of natural products which exhibit anti-infective, antitumor, immunosuppressive, and cholesterol-lowering properties, among others [1]. The modular architecture of polyketide synthases provides an attractive scaffold for biosynthetic engineering [2]. However, many natural products from this family require post–polyketide synthase modifications, including glycosylation, alkylation, and oxidation/reduction, to be fully active [3]. For example, glycosylation plays a critical role in the activity of macrolide antibacterial agents, such as erythromycin. Reconstitution of glycosylation pathways from soil bacteria into heterologous hosts requires the horizontal transfer of genes that encode nucleoside diphosphate (NDP) sugar biosynthesis, including aminosugars and deoxysugars, as well as appropriate glycosyl transferases capable of ligating these activated sugars to acceptor substrates. Because of these challenges, there are very few examples of reconstitution of glycosylation pathways in heterologous hosts [4–6]. In *Escherichia coli,* for example, glycosylation efficiency is low [7].

**Figure 1:**
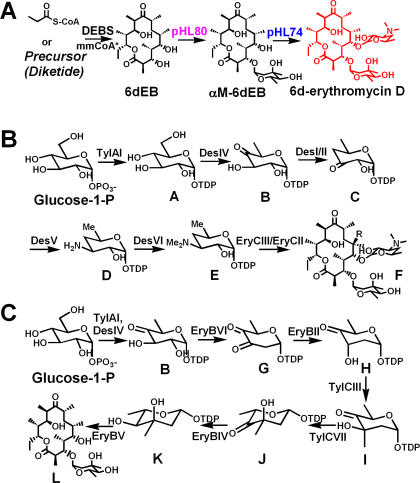
Production of 6d-EryD in E. coli (A) Biosynthesis of 6d-EryD in E. coli BAP1. (*mmCoA, methylmalonyl-CoA). (B) Reconstitution of the TDP-desosamine biosynthesis and transfer pathway (pHL74, pHL50). A, TDP-D-glucose; B, TDP-4-keto-6-deoxy-D-glucose; C, TDP-3-keto-4,6-dideoxy-D-glucose; D, TDP-3-amino-4,6-dideoxy-D-glucose; E, TDP-D-desosamine; F, R = OH, erythromycin D, R = H, 6-deoxyerythromycin D. (C) Reconstitution of the TDP-mycarose biosynthesis and transfer pathway (pHL80, pHL71). G, TDP-3,4-diketo-2,6-dideoxy-D-glucose; H, TDP-4-keto-2,6-dideoxy-D-glucose; I, TDP-4-keto-3-methyl-2,6-dideoxy-D-glucose; J, TDP-4-keto-3-methyl-2,6-dideoxy-L-glucose; K, TDP–L-mycarose; L, αMEB.

Earlier work from our laboratory led to the reconstitution of the deoxyerythronolide B synthase (DEBS) pathway in *E. coli,* resulting in substantial production of the aglycone 6-deoxyerythronolide B (6dEB; ∼ 200 mg/l) [8]. The antibiotic erythromycin D, however, bears two deoxysugars, desosamine and mycarose, both of which are crucial for high antibacterial activity. Here, we describe the coexpression of both the glycosylation pathways and DEBS, resulting in successful production of biologically active 6-deoxyerythromycin D (6d-EryD) in E. coli BAP1 (Figure 1A). The reconstitution of erythromycin biosynthesis in E. coli provides a unique opportunity for a genetics-led approach to biosynthetic engineering. As a first step toward realizing this potential, an activity-based screening assay was developed. Initial applications of this high-throughput assay are also described.

## Results/Discussion

### Reconstitution of TDP-Desosamine Biosynthesis and Glycosyl Transfer

The aminosugar desosamine plays a critical role in macrolide activity as evidenced by oleandomycin, pikromycin, narbomycin, tylosin, and erythromycin antibiotics [9]. Specifically, it promotes ribosomal binding through a combination of hydrogen bonding and electrostatic interactions [10]. The biosynthesis of thymidine diphosphate (TDP)-D-desosamine (E) (Figure 1B) requires the activation of D-glucose-1-phosphate to TDP-D-glucose (A) by TDP-D-glucose synthetase, followed by dehydration at C4 and C6 via TDP-D-glucose 4,6-dehydratase [11]. Due to its high expression levels in *E. coli,* TylAI, a TDP-glucose synthetase from the tylosin gene cluster in Streptomyces fradiae [12–14], was recruited for desosamine generation. For similar reasons, DesIV, encoded by the pikromycin gene cluster from *S. venezuelae,* was used as the TDP-glucose 4,6-dehydratase [11]. When expressed in *E. coli,* these two proteins produced the expected TDP-4-keto-6-deoxy-glucose (B) from glucose-1-phosphate [11], as verified by high-performance liquid chromatography analysis in the in vitro assay (Figure S7).

The pikromycin biosynthetic enzymes, DesI and DesII, were used to catalyze C4-deoxygenation and C3-oxidation of TDP-4-keto-6-deoxy-D-glucose (B) [11,15]. DesI, a pyridoxamine-5-phosphate–dependent 4-aminotransferase, converts TDP-4-keto-6-deoxy-D-glucose (B) into TDP-4-amino-6-deoxy-D-glucose. DesII, a member of the radical S-adenosylmethionine (SAM) superfamily, harbors a [4Fe-4S] cluster that carries out the C4 deamination of TDP-4-amino-6-deoxy-D-glucose to produce TDP-3-keto-4,6-dideoxy-D-glucose (C) [11,16]. Therefore, we coexpressed the flavodoxin and flavodoxin reductase genes from E. coli as an additional electron carrier system [17,18], which resulted in increased DesI/DesII activity in vivo (as judged by complementation experiments; data not shown). The final steps of TDP-D-desosamine biosynthesis in E. coli are catalyzed by DesV, an aminotransferase catalyzing the conversion of intermediate TDP-3-keto-4,6-dideoxy-D-glucose (C) to TDP-3-amino-4,6-dideoxy-D-glucose (D) [19], and DesVI, a S-adenosylmethionine–dependent N,N′-dimethylase acting on the C3 amino group of TDP-3-amino-4,6-dideoxy-D-glucose (D) [20].

Once TDP-D-desosamine (E) is synthesized in vivo, it must be transferred to the acceptor substrate by an appropriate glycosyl transferase. Glycosyl transferases play important roles in a variety of biological processes, including cell wall biosynthesis, signal transduction, and macrolide biosynthesis [21]. Two desosaminyl transferase genes, *eryCIII* and *desVII,* that have been functionally expressed in E. coli [22–24], were evaluated. EryCIII, a desosaminyl transferase from Saccharopolyspora erythraea [22,23], catalyzes the attachment of desosamine to α-mycarosyl-erythronolide B (αMEB). The activity of this highly selective transferase increases dramatically in the presence of EryCII [23]. DesVII from S. venezuelae [25] also requires a chaperone protein, DesVIII, for full activity [24], but unlike EryCIII, it displays broad substrate tolerance for both aglycones [25] as well as TDP-sugar substrates [26]. However, since EryCII/EryCIII are more efficient in accepting the substrate of interest, αMEB, than DesVII/DesVIII (data not shown), *eryCII* and *eryCIII* were combined with the above desosamine biosynthetic genes on a single expression plasmid, pHL74 (Cm^R^) or, alternatively, pHL50 (Kan^R^) (Figure 1B and Table 1).

**Table 1:**
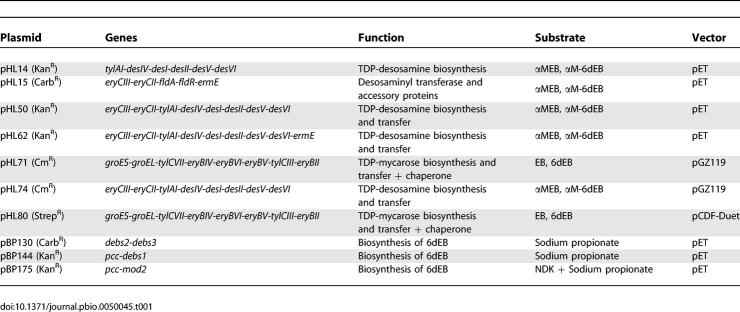
Plasmids Used

### Reconstitution of TDP-Mycarose Biosynthesis and Transfer

Mycarose is a common 2,6-deoxysugar found in polyketide compounds and contributes significantly to the high antibacterial activity of erythromycin. For example, desosaminyl clarithronolide, which lacks a mycarose substituent, has less than 2% of the activity of erythromycin D against Bacillus subtilis (unpublished data). Therefore, to synthesize fully active erythromycin analogs, we reconstituted an optimal set of TDP-L-mycarose biosynthetic genes from the homologous erythromycin and tylosin biosynthetic gene clusters (Figure 1C).

The first two steps from glucose-1-phosphate to 4-keto-6-deoxy-D-glucose (B) [12] are shared with the TDP-D-desosamine biosynthetic pathway. To synthesize TDP-L-mycarose (K) from TDP-4-keto-6-deoxy-D-glucose (B), a synthetic operon comprised of the *eryBVI, eryBII, tylCIII, tylCVII,* and *eryBIV* genes was constructed (pHL71; Table 1). The *eryBV* mycarosyl transferase gene was also included in this operon. Again, genes from the tylosin pathway were harnessed from sources that expressed well in *E. coli.* EryBVI and EryBII, which catalyze C2-deoxygenation of TDP-4-keto-6-deoxy-D-glucose (B) to yield TDP-4-keto-2,6-dideoxy-D-glucose (H), are structurally and functionally distinct from the corresponding enzymes catalyzing the C-3 deoxygenation in the biosynthesis of 3,6-dideoxysugars [27]. TylCIII, a 46.4-kDa C-methyl transferase, catalyzes C-3 methylation of the 2,6-dideoxysugar (H) to produce TDP-4-keto-3-methyl-2,6-dideoxy-D-glucose (I), and uses S-AdoMet as a cosubstrate [28]. C5-epimerization is catalyzed by TylCVII [29], whereas EryBIV, a 4-ketoreductase, converts TDP-4-keto-3-methyl-2,6-dideoxy-L-glucose (J) to TDP-L-mycarose (K) [12]. EryBV, which ordinarily transfers the mycarose sugar onto erythronolide B, is also capable of recognizing 6dEB as a substrate. Finally, plasmid pHL71 also included *E. coli groEL* and *groES* genes, as they were found to enhance glycosyl transferase gene expression in earlier studies [22]. As shown below, along with the genes responsible for desosamine biosynthesis and transfer, the entire mycarose operon functions efficiently in E. coli BL21(DE3).

### Efficiency of Deoxysugar Biosynthesis and Transfer in E. coli

**Figure 2:**
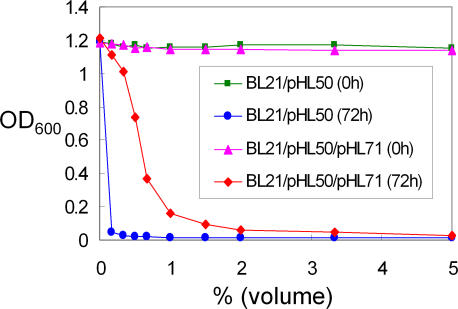
Production of 6d-EryD Blue circles, αMEB conversion to erythromycin D by BL21/pHL50. Red diamonds, 6dEB conversion to 6d-EryD by BL21/pHL50/pHL71. Cells were induced at 20 °C with 10 μM IPTG, and samples were collected 72 h after induction. αMEB or 6dEB was added at a final concentration of 100 mg/l. The abscissa (%) indicates the volume of E. coli culture medium added to LB inoculated with *B. subtilis.*

To assess the metabolic capacity of the desosamine biosynthesis and transfer pathway in *E. coli,* BL21(DE3)/pHL50 was grown in the absence of antibiotics. The IPTG-induced culture was fed with αMEB (isolated from a mutant strain of S. erythraea), and the time course of erythromycin D accumulation was monitored by a B. subtilis inhibition assay using an authentic sample of erythromycin D as a reference (Figure S1A). Upon induction with 10 μM IPTG and the addition of 100 mg/l αMEB at 20 °C, approximately 25% conversion to erythromycin D was observed (Figure 2). Consistent with earlier observations [22], the biosynthetic efficiency doubled when GroEL/GroES were coexpressed with the synthetic desosamine operon.

The cumulative efficiency of the mycarose and desosamine pathways was evaluated in shake-flask studies with E. coli BL21(DE3)/pHL71/pHL50, using 100 mg/l 6dEB substrate. After 72 h, the culture supernatant showed activity comparable to 3 mg/l erythromycin D (Figure 2 and Figure S1B). Because 6-deoxyerythromycins are less active than the corresponding erythromycins [30,31], the biosynthetic efficiency of the two-sugar pathway was judged to be >10 mg/l.

### Macrolide Resistance and Export

Macrolide antibiotics inhibit bacterial cell growth by binding to the exit tunnel of the 50S ribosomal subunit [32]. A common resistance mechanism involves ribosome methylation, which prevents macrolide binding to the ribosome by introducing steric hindrance in the antibiotic binding pocket. Although the heterologous expression of the methylase gene *ermE* [33] in E. coli BL21(DE3) renders the host more resistant to erythromycin A in liquid culture (unpublished data), on semisolid plate media the host is not inhibited by endogenously produced erythromycin in the absence of *ermE.* Correspondingly, BL21(DE3)/pHL50 is naturally resistant to αMEB up to 400 mg/l, and *ermE* was not deemed necessary in our system.

It is known that endogenous multidrug pumps such as MacAB [34] and AcrAB [35,36] in E. coli are efficient at exporting macrolide antibiotics bearing the mycarose sugar [34,37]. To test whether αMEB is secreted by BL21/pHL80/pHL50, we cospotted E. coli strains BL21(DE3)/pHL80/pHL50 (pHL80 is a Strep^R^ analog of the mycarose plasmid pHL71) and BL21(DE3)/pHL50 in close proximity on a Petri plate containing 6dEB. It was anticipated that αMEB secreted by the former strain would be converted into 6d-EryD by the latter strain. A dramatic increase in antibiotic activity was observed around BL21(DE3)/pHL50 (Figure S2). This is consistent with the observations that monoglycosylation is efficient, whereas diglycosylation is inefficient (Figure 2). It also suggests that, while endogenous multidrug resistance mechanisms in E. coli enable this host to synthesize erythromycin without self-destruction, they also contribute to biosynthetic inefficiency by prematurely exporting the mycarosylated precursor.

### Production of 6d-EryD in E. coli

To synthesize a bioactive erythromycin in *E. coli,* the host strain BAP1 (engineered for the phosphopantetheinyl modification of DEBS as well as propionyl-CoA biosynthesis [38]) was cotransformed with plasmids pBP144 (Carb^R^, encoding the DEBS1 and the *pccAB* genes), pBP130 (Kan^R^, encoding for DEBS2 and DEBS3), pHL74 (a Cm^R^ analog of the desosamine plasmid pHL50), and pHL80 (a Strep^R^ analog of the mycarose plasmid pHL71). The resulting strain produced low levels of 6d-EryD, detectable by mass spectrometry, but estimated to be very low. This poor productivity is consistent with an earlier report of < 1 mg/l glycosylated macrolide production in E. coli [7]. Indeed, it was not possible to observe bioactivity from single colonies of this transformant on a Petri plate. However, as described below, an activity-based screening assay was developed to enhance the productivity of this progenitor strain of *E. coli.*

### Bioactivity-Based Screening Assay for 6d-EryD Overproducers

**Figure 3:**
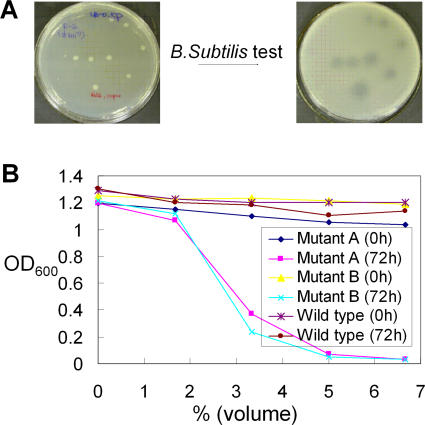
Screening for 6d-EryD Overproducers (A) Single-colony screening assay of the Mutant A: B. subtilis inhibition assay after 60 h. (B) Shake-flask production of 6d-EryD by mutants, as assayed via B. subtilis inhibition assay. Comparison of Mutant A versus Mutant B versus wild-type of strain BAP1/pBP130/pBP144/pHL80/pHL74 after 72 h. Cells were grown in the presence of 2 g/l propionate, and induced with 0.5 mM IPTG at 30 °C.

Although single colonies of E. coli BAP1/pBP144/pBP130/pHL80/pHL74 were unable to synthesize adequate antibiotic to generate a halo in a B. subtilis overlay assay, small patches (∼0.5 cm^2^) of individual colonies revealed detectable growth inhibition in an equivalent assay. We therefore tested several independent transformants via this method and isolated single colonies of the best two producers by restreaking, followed by repeated bioassays on small patches derived from individual colonies. After three rounds of screening, individual colonies showed a readily observable signal in a B. subtilis overlay assay (Figure 3A). Two overproducers, mutant A and mutant B, produced 6d-EryD, comparable to 2 mg/l erythromycin D in shake flask experiments (Figure 3B). Considering the effect of 6-hydroxyl group on the activity of erythromycin, we estimated a titer of 5–10 mg/l 6d-EryD production in shake-flask experiments.

### Analysis of 6d-EryD Overproducers

**Figure 4:**
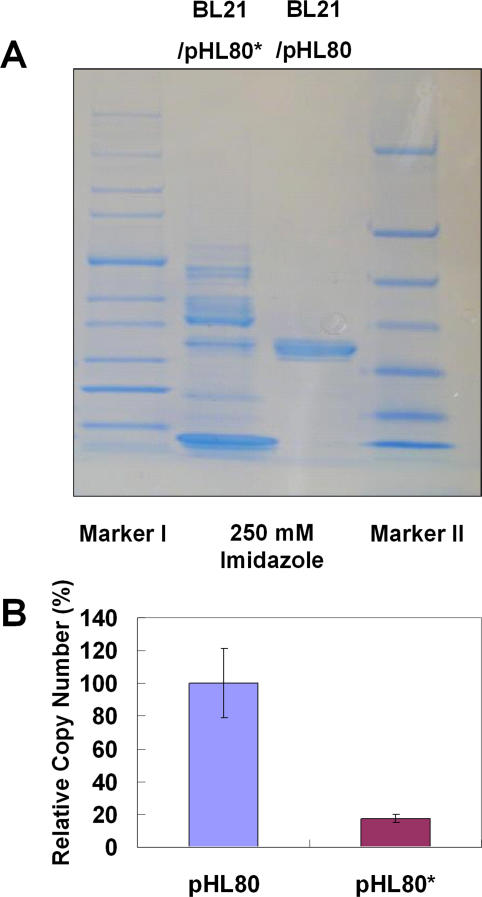
Analysis of the Mechanistic Basis for 6d-EryD Overproduction by Mutant A (A) SDS-PAGE analysis of Ni affinity-purified lysates of BL21/pHL80* and BL21/pHL80 following induction with 0.5 mM IPTG at 30 °C for 5 h. (B) The relative copy number of pHL80 and pHL80* in E. coli XL1-Blue.

To obtain preliminary insights into the mechanistic basis for 6d-EryD overproduction in the above mutants, we first compared the stability of each plasmid of a representative overproducer and the wild-type strain. Although the overproducer showed marginally improved stability (∼2×), this difference could not explain the considerable increase in antibiotic productivity. Therefore, we purified each plasmid from a mutant cell line and retransformed E. coli BAP1 cells along with the other three wild-type plasmids. Only the mutant plasmid pHL80* was sufficient to reconstitute the overproducer phenotype, as judged by single-colony assays (Figure S3). Restriction analysis of pHL80* showed no differences relative to pHL80 that would be suggestive of a subtle mutation. However, comparative protein expression analysis of BL21(DE3)/pHL80* versus BL21(DE3)/pHL80 showed major differences after 5 h of induction with 0.5 mM IPTG at 30 °C (Figure 4). The mutant pHL80* revealed more balanced expression of the mycarose biosynthetic and transfer enzymes compared to pHL80, which selectively overexpressed the ketoreductases EryBII and EryBIV. Further analysis revealed that the copy number of pHL80* is significantly lower than pHL80 (15%–20%; Figure 4B). Other investigators have also observed that lower-copy-number plasmids can enhance the production of natural products in bacteria [39]. Presumably, the lower copy number of pHL80* can reduce the overall burden of heterologous protein expression in the host cell, although further investigations are warranted in this regard.

### Bioassay-Guided Evolution of Precursor-Directed Biosynthesis of Antibiotics

Because the plasmid pHL80* significantly enhanced macrolide antibiotic biosynthesis in *E. coli,* we hypothesized it would also improve the productivity of other related antibiotic-producing systems. We therefore introduced pHL80* and pHL74 into BAP1/pBP130/pBP175, which contains a deletion of the loading didomain and module 1 of DEBS. The resulting strain is inherently incapable of polyketide production, but does so in the presence of a variety of exogenously introduced thioester substrates [40]. This method has been used to prepare a wide range of new macrolide antibiotics with promising biological activities [41,42]. As predicted, colonies of BAP1/pBP130/pBP175/pHL80*/pHL74 supplemented with 100 μM (2*S*,3*R*)-2-methyl-3-hydroxy-pentanoyl-SNAc (NDK) produced 6d-EryD, whereas no signal was observed with the control strain harboring wild-type plasmids (BAP1/pBP130/pBP175/pHL80/pHL74) in the B. subtilis inhibition assay. This result demonstrated the utility of pHL80* as a general toolkit for improving glycosylated macrolide biosynthesis in *E. coli.*

**Figure 5:**
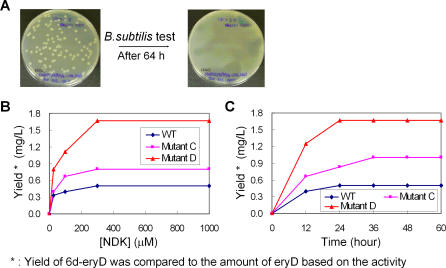
Screening of BAP1/pBP130/pBP175/pHL80*/pHL74 for Mutants (e.g., Mutant C, Mutant D) Capable of Improved Precursor-Directed Biosynthesis (A) Single-colony screening assay of BAP1/pBP130/pBP175/pHL80*/pHL74 with 0.5 mg/ml propionate and 100 μM synthetic diketide substrate (NDK). (B) Kinetics of Mutant C, Mutant D, and wild-type. BAP1/pBP130/pBP175/pHL80*/pHL74 in shake-flask experiments with fixed growth time (48 h), and with different [NDK]. (C) Kinetics of Mutant C, Mutant D, and wild-type. BAP1/pBP130/pBP175/pHL80*/pHL74 in shake-flask experiments with fixed [NDK] (300 μM), and at different timepoints. (In shake-flask experiments, the cultures were induced by adding 0.1 mM IPTG at 30 °C with 2.5 g/l propionate and NDK.)

Single colonies of E. coli BAP1/pBP130/pBP175/pHL80*/pHL74 were grown for 48–60 h in the presence of 25 μM, 100 μM, 300 μM, or 1 mM NDK. At substrate concentrations below 100 μM, halo sizes increased with increasing NDK concentrations, whereas no further increase in signal was observed at [NDK] > 100 μM (unpublished data). Therefore, we screened colonies at a subsaturating NDK concentration of 25 μM, and isolated mutants (Mutant C, Mutant D) that exhibited a significantly larger halo size (Figure 5A). Shake-flask comparisons of Mutant C, Mutant D, and wild-type BAP1/pBP130/pBP175/pHL80*/pHL74 revealed that Mutant C and Mutant D are more effective than wild-type in converting NDK to 6d-EryD (Figure 5B and 5C). Although the mechanistic basis for this phenotype is under investigation, this example illustrates the potential for directed evolution in macrolide biosynthetic engineering.

In conclusion, we have reconstituted the 6d-EryD biosynthetic pathway in *E. coli,* and used it to develop an activity-based screen for macrolide biosynthesis. Our results represent the first example of the bioassay-guided evolution of an antibiotic pathway in a heterologous host, thereby opening the door for harnessing the power of genetics for understanding and manipulating polyketide biosynthesis.

## Materials and Methods

### Construction of plasmids.

*eryBII, eryBIV, eryBV, eryBVI, eryCII,* and *ermE* were amplified from S. erythraea genomic DNA by PCR, using primers with NdeI and SpeI restriction sites (underlined): *eryBIV,* forward, 5′-AAAAAACATATGAATGGGATCAGTGATTCCCCGCGT-3′, reverse, 5′-AAAAAAACTAGTGTGCTCCTCGGTGGGGGT-3′; *eryBVI,* forward, 5′- AAAAAACATATGCGGGTCTTGATCGACAACGCC-3′, reverse, 5′-AAAAAAACTAGTTCCGGCGGTCCTGGTGTA-3′; *eryBV,* forward, 5′-AAAAAACATATGCGGGTACTGCTGACGTCCTTC-3′, reverse, 5′-AAAAAAACTAGTGCCGGCGTGGCGGCG-3′; *eryBII,* forward, 5′-AAAAAACATATGACCACCGACGCCGCGAC-3′, reverse, 5′-AAAAAAACTAGTCTGCAACCAGGCTTCCGGC-3′; *eryCII,* forward, 5′- AAAAAACATATGACCACGACCGATCGCGCC-3′, reverse, 5′- AAAAAAACTAGTTCAGAGCTCGACGGGGCA-3′; and *ermE,* forward, 5′- AAAAAACATATGAGCAGTTCGGACGAGCAGCCG-3′, reverse, 5′- AAAAAAACTAGTCCGCTGCCCGGGTCCGCC-3′.

Genes encoding GroEL and GroES were amplified from E. coli BL21(DE3) genomic DNA using following primers with NdeI and SpeI restriction sites (underlined) by PCR: GroES, forward, 5′-AAAAAACATATGAATATTCGTCCATTGCATGATCG-3′, reverse, 5′-AAAAAAACTAGTTTACGCTTCAACAATTGCCAGAA-3′; and GroEL, forward, 5′- AAAAAACATATGGCAGCTAAAGACGTAAAATTCGGT-3′, reverse, 5′-AAAAAAACTAGTTTACATCATGCCGCCCATGC-3′.

Genes encoding *tylAI, tylCIII,* and *tylCVII* were amplified from genomic DNA of S. fradiae using following primers with restriction sites (underlined) by PCR: *tylAI,* forward, 5′- AAAAAACATATGAACGACCGTCCCCGCCGC-3′, reverse, 5′-AAAA AACTCGAGTCACTGTGCCCGGCTGTC-3′; *tylCIII,* forward, 5′-AAAAAACATATGCCCGCTGTTCCCCGAGAG-3′, reverse, 5′-AAAAAAACTAGTTACGACGTCGAGCCGGGG-3′; and *tylCVII,* forward, 5′-AAAAAACATATGATCATCACCGAGACCAGGGTC-3′, reverse, 5′-AAAAAACTCGAGCATGGCCGGATAGGCC −3′.

Each PCR product was cloned into pET28 or pET21 vectors. Genes were coexpressed as synthetic operons. Specifically, *eryCIII, eryCII, tylAI, desIV, desI, desII, desV,* and *desVI* were cloned into a modified pET28 vector using XbaI and SpeI restriction sites, yielding pHL50. Similarly, pHL71 was constructed by inserting *groEL, groES*, *tylCVII, eryBIV, eryBVI, eryBV, tylCIII,* and *eryBII* genes into a modified pGZ119 vector.

### Bacterial strains.

For cloning purposes, E. coli XL1-Blue strain was used. For gene expression, E. coli BL21(DE3) was used. Alternatively, for the production of 6d-EryD, E. coli BAP1 [38] was cotransformed with pBP130, pBP144, pHL80, and pHL74.

### Conversion of αMEB into erythromycin D.

BL21/pHL50 (200 ml) was grown at 37 °C to an OD_600_ = 0.6. The culture was chilled on ice for 10 min and spun down at 4,000*g* for 15 min. After washing with LB, the cell pellet was resuspended in 10 ml of fresh LB without any antibiotic. To this culture, 100 mg/l αMEB and IPTG was added, and the cell culture was incubated at 18 °C or 20 °C for 72 h.

### Conversion of 6dEB into 6d-EryD.

BL21(DE3) cells were cotransformed with pHL50 and pHL71 and grown in the presence of kanamycin (50 μg/ml) and chloramphenicol (34 μg/ml) at 37 °C. A 100-ml LB culture was shaken at 200 rpm at 37 °C until OD_600_ = 0.6. The culture was chilled on ice for 10 min and cells were harvested by spinning 10 min at 4,000*g.* Cells were washed once with 100 ml of ice-cold LB, resuspended in 5 ml of LB without antibiotics, and induced with 10 μM IPTG in the presence of 100 μg/ml 6dEB at 20 °C.

### B. subtilis inhibition assays.

To detect or quantify a glycosylated macrolide in the spent culture medium of an E. coli strain, a test sample of the 0.2 μm filtered culture medium was added to a freshly inoculated culture of *B. subtilis.* No exogenous antibiotics were used during the growth of the E. coli culture. The growth rate of *B. subtilis,* calculated by measuring OD_600_ as a function of time, was used to estimate the amount of macrolide antibiotic. To detect macrolides produced by single E. coli colonies, LB plates were prepared with 0.5 mg/ml of sodium propionate or other substrates, such as 6dEB or αMEB, added at appropriate concentrations. A sterilized cellophane disk soaked with water was placed on top of the LB plate. The test strain of E. coli was plated on the cellophane disk at an appropriate cell density. After 2–3 d at 30 °C, the cellophane disk was removed from the plate, and 2.5 ml of a soft agar overlay containing 0.1% B. subtilis culture was added to each plate. After overnight growth at 30 °C, halos arising due to growth inhibition of B. subtilis were visualized around individual colonies.

### Biosynthesis of 6d-EryD.

E. coli BAP1 cells were cotransformed with pBP130 (Carb^R^), pBP144 (Kan^R^), pHL80 (Sm^R^), and pHL74 (Cm^R^). Cells were grown in 1 l LB with antibiotics until OD_600_ = 0.6, and concentrated in 50 ml of LB in shake-flask as above without any antibiotics, and induced with 2.5 g/l sodium propionate and 0.1 mM IPTG at 20 °C or 30 °C.

### Analyses of Mutant A.

To isolate individual plasmids from the overproducer Mutant A, a 10-ml LB culture was grown overnight with kanamycin (50 mg/l), carbenicillin (100 mg/l), chloramphenicol (34 mg/l), and streptomycin (50 mg/l). The cells were centrifuged, and plasmids were purified by ethanol precipitation. The purified plasmid mixture (1 μl) was transformed to XL1-Blue and spread on LB plates with each of the four antibiotics present individually. The antibiotic resistance profiles of selected colonies from each plate were screened, and individual plasmids were purified from colonies bearing only one plasmid.

To test the stability of each plasmid, mutant A was grown in LB with all antibiotics (kanamycin, carbenicillin, chloramphenicol, and streptomycin), and diluted 10^6^-fold. 100-μl aliquots were spread on plates containing each antibiotic individually. By comparing the number of colonies on each plate to a control plate with no antibiotic, the stability of each plasmid was assessed.

To test whether the plasmids in Mutant A had undergone gross structural changes, appropriate restriction digests were analyzed for each plasmid (pBP130: XmnI, NotI; pBP144: XhoI, NdeI + EcoRI; pHL80: NotI, XhoI; pHL74: SphI, EcoRI). The expected fragments were verified by agarose gel electrophoresis.

To evaluate protein expression levels in BL21(DE3)/pHL80* and BL21(DE3)/pHL80, 100-ml LB cultures with 50 μg/ml streptomycin were incubated at 37 °C until OD_600_ = 0.6. The culture was induced with 0.5 mM IPTG at 30 °C, and allowed to incubate for 5 h. Cells were harvested by centrifugation at 4,000*g,* and lysed by sonication. Ni-NTA affinity purification was used for further enrichment of proteins expressed by plasmid-borne genes.

To analyze the relative copy number of pHL80 and pHL80* in *E. coli,* 5-ml LB cultures were grown with 50 μg/ml streptomycin at 37 °C. After 12 h, cells were harvested, and DNA was extracted using QIAprep Spin Miniprep Kit (DNA was eluted after 2 min of incubation with 200 μl of 70 °C H_2_O; QIAGEN, http://www.qiagen.com). The amount of DNA was calculated based on the absorbance at 260 nm, and the cell density was calculated by serial dilution. The relative copy number of a plasmid was measured as the amount of plasmid DNA per cell.

### Precursor-directed biosynthesis with BAP1/pBP130/pBP175/pHL80(*)/pHL74.

The same procedure used in 6dEB or αMEB feeding experiments was also used in these experiments, except that 2.5 mg/ml propionate and an appropriate concentration of NDK were added instead of 6dEB or αMEB. In shake-flask experiments, the culture was induced by 0.1 mM IPTG at 30 °C.

## Supporting Information

Figure S1 Production of 6d-EryD: B. subtilis Growth Inhibition Assay (A) Calibrating the antibacterial activity of authentic erythromycin D.
(B) 6dEB conversion to 6d-EryD by BL21(DE3)/pHL50/pHL71. Antibiotic concentration was measured by a B. subtilis inhibition assay. Protein expression was induced at 20 °C with 10 μM IPTG, and a sample was taken 72 h after induction. 6dEB was added at a final concentration of 100 mg/L. The abscissa (%) indicates the volume of E. coli culture medium added to LB inoculated with *B. subtilis.*
(591 KB TIF)

Figure S2 Bioassay Detection of αMEB Exported from BL21(DE3)/pHL80/pHL50 A converter strain, BL21(DE3)/pHL50, harboring the desosamine biosynthetic and transfer genes, was streaked around BL21/pHL80/pHL50 on a plate containing 5 mg/l 6dEB. The B. subtilis overlay test was performed at 30 °C after 65 h.
(9.4 MB TIF)

Figure S3 Analysis of Mutant A: Identification of Overproducing Mutation on pHL80* (A) Transformants derived from wild-type pBP130, wild-type pBP144, mutant A–derived pHL80, and mutant A–derived pHL74.
(B) Transformants derived from wild-type pBP130, wild-type pBP144, mutant A–derived pHL80, and wild-type pHL74.
(C) Transformants derived from wild-type pBP130, wild-type pBP144, wild-type pHL80, and mutant A–derived pHL74.
The LB plates (with 0.5 mg/ml propionate) were grown at 30 °C for 60 h before being overlaid with the B. subtilis tester strain.
(8.7 MB TIF)

Figure S4 LC-MS analysis of 6d-EryD production by BAP1/pBP130/pBP144/pHL80*/pHL74 The culture was induced with 100 μM IPTG in the presence of 2.5 g/L propionate at 30 °C for 72 h.
(A) Base peak (positive ion electrospray ionization) full-spectrum (MW 200–2,000), total ion current (TIC) = 3.72 × 10^7^, 1 μg of erythromycin A was added as an external standard.
(B) 6d-EryD, RT (retention time): 15.02–15.20 min, TIC = 1.54 × 10^7^.
(1.1 MB TIF)

Figure S5 Analysis of 6d-EryD by High-Resolution Mass Spectrometry (A) Analysis of 6d-EryD from shake-flask cultures of BAP1/pBP130/pBP144/pHL80/pHL74 Mutant A.
(B) Analysis of 6d-EryD from shake-flask cultures of BL21/pHL50/pHL71 with 100 mg/l 6dEB.
(3.2 MB TIF)

Figure S6 Negative Control Experiments for Glycosylations Negative controls for the experiments in Figure 2.
(862 KB TIF)

Figure S7 Conversion of TDP-Glucose to TDP-4-Keto-6-Deoxy-Glucose by DesIV The assays were performed in 100 mM phosphate (pH 8.0), 1 mM DTT, and 5% glycerol in the presence of 1 mM TDP-D-glucose, 1 mM NAD^+^, and 8 μM DesIV at 25 °C and monitored by UV detector at 264 nm.
For high-performance liquid chromatography analysis, H_2_O with 0.1% TFA and acetonitrile with 0.1% TFA were used as buffer A and buffer B. The gradient program used was 2% buffer B for 2 min, 2%–16% buffer B for 10 min, and 95% buffer B for 5 min. Alltech Apollo C18 column, 25 mm × 4 mm (http://www.alltech.com), was used.
(892 KB TIF)

## Abbreviations

6dEB: 6-deoxyerythronolide B
6d-EryD: 6-deoxyerythromycin D
αMEB: α-mycarosyl-erythronolide B
DEBS: deoxyerythronolide B synthase
NDK: (2S,3R)-2-methyl-3-hydroxy-pentanoyl-SNAc
